# Supplementary material for: Synthesis and Design of Hybrid Metalloporphyrin Polymers Based on Palladium (II) and Copper (II) Cations and Axial Complexes of Pyridyl-Substituted Sn(IV)Porphyrins with Octopamine
Source: Polymers (Basel). 2023 Feb 20;15(4):1055. doi: 10.3390/polym15041055 (PMC9959591; doi:10.3390/polym15041055)
Supplement: Supplementary file 1 [file polymers-15-01055-s001.zip › polymers-2212909-supplementary.pdf]

Supplementary Information

of the article

Synthesis and design of hybrid metalloporphyrin polymers based on palladium (II) and copper (II) cations and axial complexes of pyridyl-substituted Sn(IV)porphyrins with octopamine

Anastasia E. Likhonina, Galina M. Mamardashvili, Ilya A. Khodov, Nugzar Z. Mamardashvili*

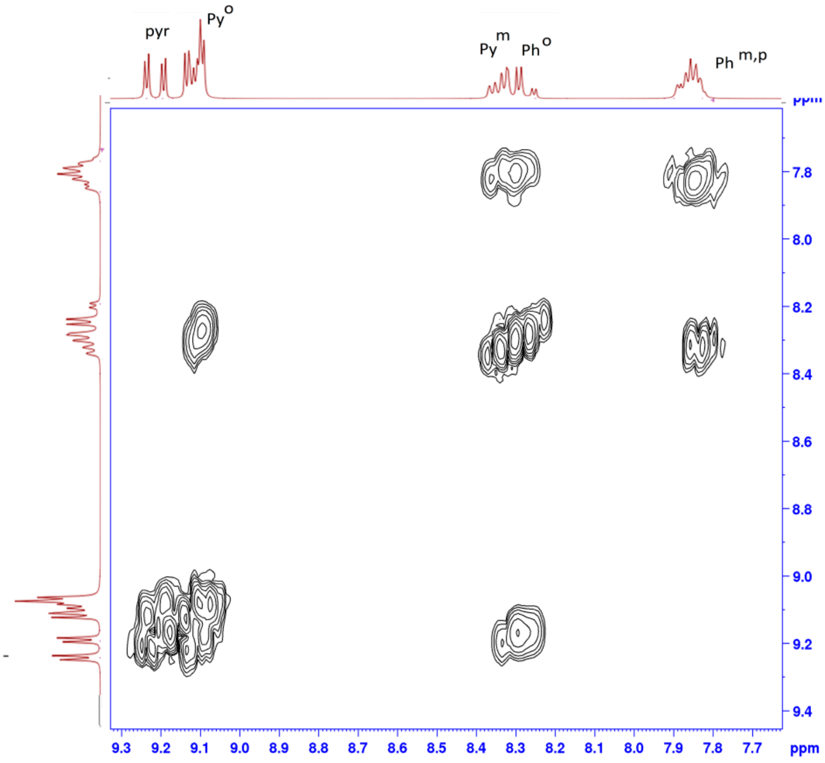

Figure S1. Partial ^1H-^1H COSY of the $Sn(OH)_2P$ in DMSO-d_6

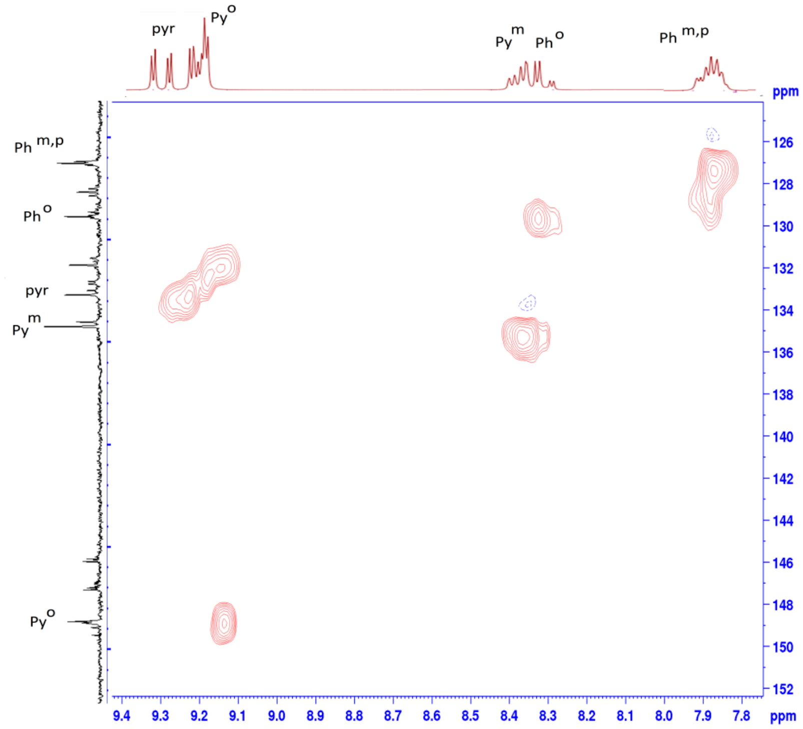

Figure S2. Partial ^{1}H-^{13}C HSQC of the Sn(OH)$_2$P in DMSO-d_6

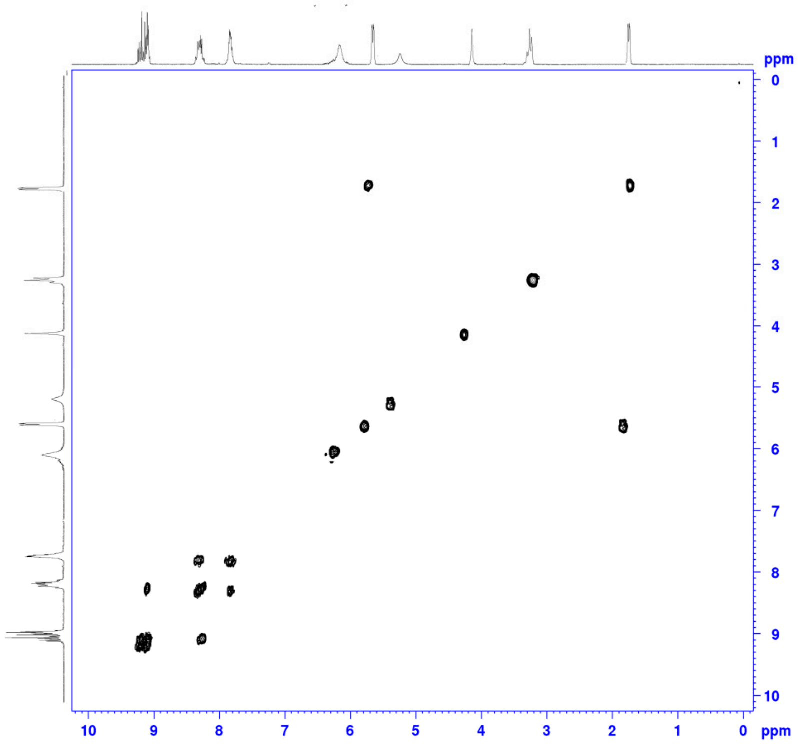

Figure S3. ^{1}H-^{1}H COSY of the Sn(L)$_2$P

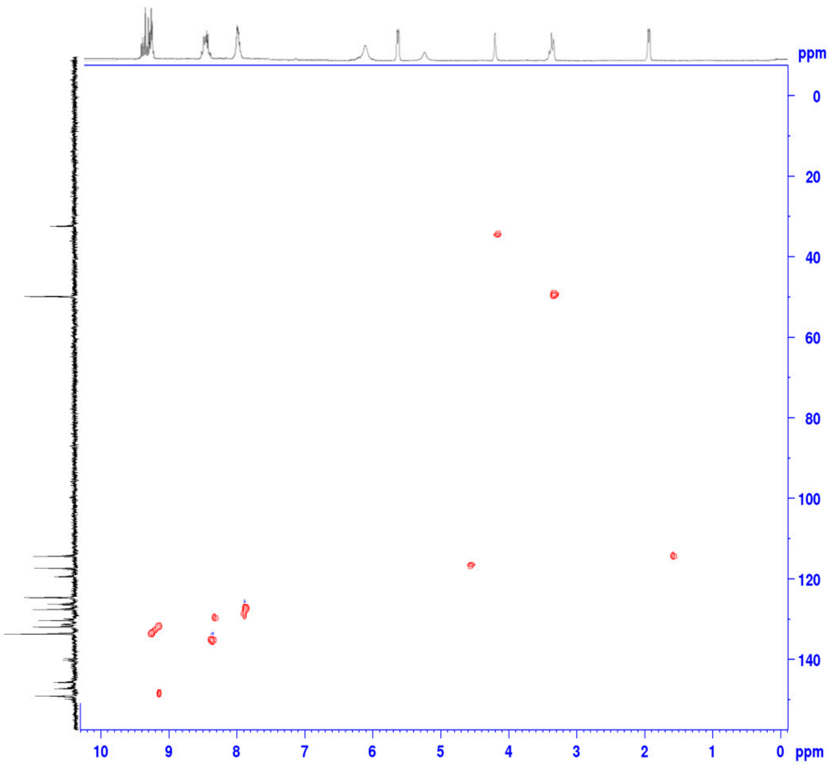

Figure S4. ^{1}H-^{13}C HSQC of the $Sn(L)_2P$

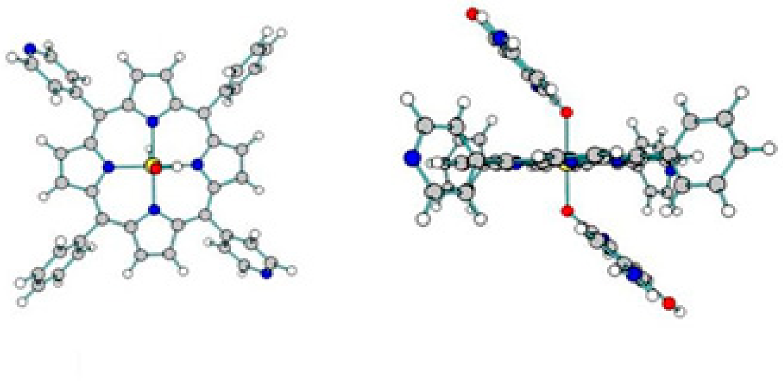

1 **6**

Figure S5. Optimized structures of the $Sn(OH)_2P$ **1** and $Sn(L)_2P$ **6** complexes.

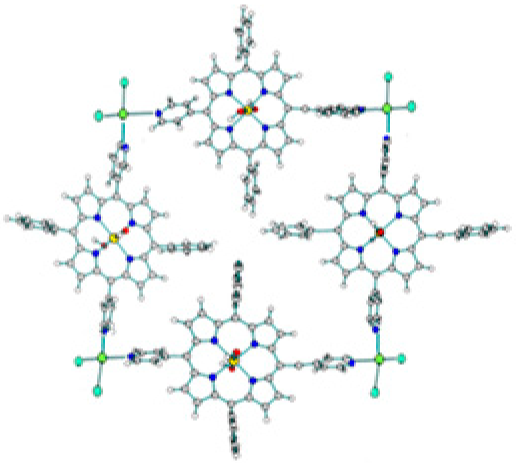

Figure S6. Cell structure of a 2D polymer - a cyclic tetramer complex (Sn(OH)2P)4(PdCl2)4, obtained by DFT with the B3LYP/3-21G kit.

Table S1. Barret-Joyner-Halenda (BJH) pore size distribution for compounds 2-4 (r is pore radius, V is pore volume, S is surface area).

Name	Adsorption			Desorption		
	r, нм	V, см^3/г	S, м^2/г	r, нм	V, см^3/г	S, м^2/г
Sn(OH)$_2$P **1**	-	-	-	1.531	0.024	32
	1.681	0.023	28	1.704	0.045	56
	1.881	0.047	53	1.905	0.069	81
	2.111	0.064	69	2.144	0.092	102
	2.386	0.088	89	2.433	0.115	121
	2.728	0.109	105	2.792	0.139	139
	3.163	0.128	116	3.237	0.159	151
	3.743	0.150	128	3.844	0.185	164
	4.559	0.173	138	4.703	0.207	174
	5.780	0.190	144	5.987	0.229	181
	7.899	0.211	150	8.145	0.255	188
	13.140	0.237	153	13.667	0.284	192
	52.879	0.269	155	53.283	0.306	193
Sn(L)$_2$P **6**	-	-	-	1.53	0.033	43
	1.69	0.025	30	1.70	0.072	89
	1.88	0.079	87	1.90	0.112	131
	2.11	0.120	126	2.15	0.180	195
	2.39	0.173	170	2.44	0.191	203
	2.74	0.215	201	2.79	0.257	251
	3.17	0.227	208	3.24	0.293	273
	3.75	0.250	221	3.84	0.329	292
	4.57	0.284	235	4.67	0.354	302
	5.80	0.319	248	5.96	0.408	321

	7.91	0.336	252	8.30	0.442	329
	12.67	0.372	258	13.67	0.479	334
	43.54	0.378	258	44.25	0.482	334
$(Sn(L)_2P)_2Cu$ **7**	1.68	0.028	34	1.70	0.033	39
	1.88	0.061	68	1.91	0.074	82
	2.11	0.100	105	2.15	0.105	110
	2.39	0.126	127	2.43	0.146	145
	2.74	0.149	144	2.79	0.180	168
	3.18	0.191	171	3.25	0.207	185
	3.76	0.214	183	3.85	0.234	200
	4.55	0.235	192	4.70	0.272	215
	5.77	0.260	200	5.98	0.299	225
	7.87	0.297	210	8.31	0.330	232
	12.70	0.316	213	13.91	0.366	237
	43.67	0.353	215	44.55	0.391	238
$(Sn(OH)_2P)_4(PdCl_2)_4$ **2**	1.694	0.501	592	1.698	0.594	700
	1.889	1.002	1122	1.899	1.019	1147
	2.120	1.623	1.708	2.134	1.657	1745
	2.409	2.075	2083	2.419	2.189	2185
	2.759	2.416	2330	2.771	2.679	2539
	3.184	2.972	2680	3.201	3.246	2893
	3.767	3.343	2877	3.819	3.684	3122
	4.588	3.888	3114	4.654	4.198	3344
	5.849	4.255	3240	5.908	4.752	3531
	8.034	4.703	3351	8.322	5.127	3621
	13.204	5.071	3407	13.503	5.573	3687
	51.174	5.415	3420	51.224	5.890	3700
$(Sn(L)_2P)_4(PdCl_2)_4$ **3**	1.687	0.310	368	1.702	0.463	544
	1.891	0.688	768	1.897	0.825	925
	2.122	1.005	1066	2.124	1.294	1368
	2.408	1.283	1297	2.412	1.647	1660
	2.763	1.655	1566	2.755	2.083	1976
	3.202	1.929	1737	3.202	2.458	2211
	3.789	2.192	1876	3.828	2.905	2445
	4.623	2.511	2014	4.645	3.279	2606
	5.861	2.857	2132	5.914	3.607	2716
	7.998	3.130	2201	8.209	4.010	2814
	13.088	3.327	2231	13.386	4.270	2853
	53.217	3.593	2241	53.366	4.588	2865
$((Sn(L\text{-}Cu)_2P)_4(PdCl_2)_4)_n$ **4**	1.697	0.016	18.5	1.697	0.043	50.8
	1.899	0.039	42.7	1.901	0.087	96.6
	2.132	0.053	56.0	2.133	0.124	132
	2.414	0.081	79.5	2.408	0.168	168
	2.770	0.118	106.2	2.745	0.208	197

	3.225	0.129	113.2	3.193	0.515	225
	3.812	0.172	135.9	3.813	0.288	244
	4.610	0.181	139.7	4.645	0.330	262
	5.885	0.204	147.4	5.978	0.350	269
	8.112	0.211	149.1	8.261	0.370	273
	12.151	0.222	150.7	13.752	0.406	279
	51.219	0.230	151.0	51.750	0.427	279
